# Assessing pre-season workload variation in professional rugby union players by comparing three acute:Chronic workload ratio models based on playing positions

**DOI:** 10.1016/j.heliyon.2024.e37176

**Published:** 2024-08-30

**Authors:** Xiangyu Ren, Simon Boisbluche, Kilian Philippe, Mathieu Demy, Xiaopan Hu, Shuzhe Ding, Jacques Prioux

**Affiliations:** a Sino-French Joint Research Center of Sport Science, Key Laboratory of Adolescent Health Assessment and Exercise Intervention of Ministry of Education, College of Physical Education and Health, East China Normal University, 200241, Shanghai, China; b Movement, Sport, Health Laboratory, Rennes 2 University, 35170, Bruz, France; c Department of Sports Sciences and Physical Education, École normale supérieure de Rennes, 35170, Bruz, France; d Rugby Club Vannes, French Rugby Federation, 56000, Vannes, France; e Laboratory of Movement, Balance, Performance and Health, University of Pau and Pays de l’Adour, Tarbes, EA-4445, France

**Keywords:** Team sport, Global positioning system, External load, ACWR, Training load

## Abstract

Quantifying the pre-season workload of professional Rugby Union players, in relation to their respective positions not only provides crucial insights into their physical demands and training needs but also underscores the significance of the acute:chronic workload ratio (ACWR) in assessing workload. However, given the diversity in ACWR calculation methods, their applicability requires further exploration. As a result, this study aims to analyze the workload depending on the player's positions and to compare three ACWR calculation methods. Fifty-seven players were categorized into five groups based on their playing positions: tight five (T5), third-row (3R), number nine (N9), center, and third line defense (3L). The coupled and uncoupled rolling averages (RA), as well as the exponentially weighted moving average ACWR method, were employed to compute measures derived from GPS data. Changes throughout the pre-season were assessed using the one-way and two-way analysis of variance. The results revealed that N9 covered significantly greater distances and exhibited higher player load compared to T5 and 3L [p < 0.05, effect size (ES) = 0.16–0.68]. Additionally, 3L players displayed the highest workload across various measures, including counts of accelerations and decelerations (>2.5 m s^−2^), accelerations (>2.5 m s^−2^), acceleration distance (>2 m s^−2^), high-speed running (>15 km h^−1^), very high-speed running (>21 km h^−1^, VSHR), sprint running (>25 km h^−1^, SR) distance. When using coupled RA ACWR method, centers exposed significantly greater values to T5 (p < 0.05, ES = 0.8) and 3R (p < 0.05, ES = 0.83). Moreover, centers exhibited greater (p < 0.05, ES = 0.67–0.91) uncoupled RA ACWR values for VHSR and SR than T5 and 3R. When comparing the three ACWR methods, although significant differences emerged in some specific cases, the ES were all small (0–0.56). In light of these findings, training should be customized to the characteristics of players in different playing positions and the three ACWR calculation methods can be considered as equally effective approaches.

## 1 Introduction

Rugby Union (RU) is an intense, collision-based sport played by fifteen players, comprising eight forwards and seven backs. The game is played in two 40-min halves, totaling 80 min of play. At a professional level, RU demands a high level of technical fitness and physical strength to withstand the high-speed running bouts and impacts. Workload quantification, essential to better understand the dose-response relationship between stress and internal responses, involves meticulously recording the demands of both training and competition [[1], [2], [3]]. This quantification can take the form of external load (the work completed by an athlete measured independently of his or her internal characteristics) [4] or internal load (all the psychophysiological responses occurring during the execution of the exercise prescribed by the coach) [5]. As of today, advances in global positioning system (GPS) technology have made it possible to measure the athlete's activity during training sessions and matches [6]. To address the reduced reliability of GPS-measured distances at high speeds, tri-axial accelerometers have been integrated to quantify external load for elite RU players. Their effectiveness has been validated, particularly in the evaluation of collisions in RU [7]. Therefore, the combination of GPS and tri-axial acceleration yields a more accurate representation of each player's performance.

The championship season for French professional RU clubs typically spans eight to ten months, with a preceding pre-season training phase lasting six to thirteen weeks. This pre-season period is characterized by concentrated, consecutive weeks of intensified training, considered a crucial window for optimizing physical adaptations to meet competitive demands [[8], [9], [10]]. Consequently, a thorough analysis of pre-season workload becomes imperative. Moreover, a single measure may not capture the entirety of external load during training, it is essential to employ a multi-faceted approach, utilizing various measures and monitoring methods.

RU players exhibit diverse physiological capacities according to their playing positions, encompassing varying levels of endurance, acceleration, deceleration, and strength capabilities, thereby enabling specific roles during a match [11]. Specifically, forwards are predominantly involved in rucks, while backs cover longer distances and engage in high-speed running [12,13]. While previous studies have commonly categorized players into forwards and backs, with limited further subdivisions [[14], [15], [16], [17]], it is noteworthy that these classifications encompass a wide array of specific playing positions, including the front row, second row, back row, scrum-half, fly-half, centers, wings, and full-back. Consequently, if position-specific training programs are to be developed, it is important to monitor workload at a subdivided level of playing positions.

The pre-season training period holds significant importance for overall success throughout the season, with players who engage in more pre-season sessions positioned to partake in a greater number of in-season matches [18]. Moreover, it is crucial that coaches and players are well versed in the rationale and potential benefits of diligent monitoring right from the onset of each pre-season [2]. While prior research has focused on external load monitoring of pre-season in Rugby League [19] and Australian Football League [20] players, as well as fitness test performance before and after pre-season period in RU [21], a comprehensive assessment of the external load borne by RU players during the pre-season phase remains unexplored.

The acute:chronic workload ratio (ACWR) is a widely used mathematical model used for workload management [22]. Originally conceptualized from the ‘training stress balance’ of Banister et al. [23], Hulin and colleagues adapted it into a ratio calculation method more recently [24]. The ACWR evaluates an athlete's preparedness status by observing difference between acute and chronic workload [25]. Acute workload can be as short as one training session, or it can be the cumulative workload over a span of two to fourteen days, typically one week in team sports, which reflects the player's fatigue [26]. Conversely, chronic workload denotes the average workload over the preceding three to six weeks, predominantly derived from the four-week average of workload, reflecting the player's fitness level [27]. The choice of acute and chronic time windows has varied, with most studies using a one-week absolute:four-week (seven:twenty-eight day) rolling average (RA) ACWR [28,29]. It is reported that when seven:fourteen and seven:twenty-one-day time windows were employed in soccer, similar associations have been observed among them and the seven:twenty-eight day [28]. Additionally, both external and internal load can be applied in ACWR computations [28]. Measures collected from GPS and triaxial accelerometers, such as distance covered in various speed zones, acceleration and deceleration efforts, are applied in this formula. When substituting internal load, the most frequently reported method is the session rating of perceived exertion.

There has been considerable discussion about the best model for calculating ACWR, with some suggestions that the RA model, while computationally simple, does not adequately account for the time effect of the workload at the end of the computation cycle, whereas exponentially weighted moving average (EWMA) can give more weight to recently performed workload [30]. Another consideration in calculating RA is whether the acute and chronic workload are mathematically coupled or uncoupled, because coupled RA never exceeds “four” due to the mathematical coupling suppressing the value. In contrast, the uncoupled RA allows the ACWR to increase unconstrained as the workload increases, thus providing a better indication of whether the workload is increasing too rapidly or reaching too high a level [31]. However, the differences between these three models in quantifying workload have not been studied [27,31].

Based on the above, this study aimed to (i) quantify and compare workload of players across five distinct playing positions over the course of an eight-week pre-season span and (ii) scrutinize the discrepancies arising from the application of coupled and uncoupled RA, EWMA ACWR methodologies in assessing external load. We hypothesized (i) discernible variations in workload and ACWR among professional RU players contingent on their playing position, and (ii) minimal disparities when employing the three ACWR calculation methods during this period.

## 2 Materials and methods

### 2.1 Subjects

Data were collected from a cohort of fifty-seven professional RU players (mean ± SD age: 25.07 ± 4.82 years; height 1.85 ± 0.09 m; body mass 102.48 ± 15.7 kg). All players were divided into one of five distinct groups designated by playing position: the tight five (T5) referred to playing positions 1, 2, 3, 4, and 5 (props, hooker, and second rows), third-row (3R) included playing positions 6, 7, and 8 (flankers and number eight), number nine (N9, scrum-half), centers consisted of playing position 10, 12, and 13 (fly-half, inside center, and outside center), third line of defense (3L) were those who played in position 11, 14, and 15 (wings and full-back). T5 and 3R are forwards; N9, centers, and 3L are backs. The data in this study were gathered through regular monitoring of the players. Therefore, obtaining specific ethics committee approval was not necessary [32]. All players were provided informed consent, aligning with the principles outlined in the Declaration of Helsinki. The study protocol was executed with the assistance of the medical and technical staff of the professional team, and approved by the Ethics Committee of Rennes 2 University (approval number 2024-025). Whilst maintaining ethical rigor, the dataset was screened as to exclude players whose training had been interrupted due to injury, thereby ensuring that the analysis solely involved data from participants who had completed the entirety of the prescribed rugby training sessions.

### 2.2 Procedures

The team coaches were responsible for both prescribing and implementing the training program. Each weekly training sessions included warm-up, technical and tactical drills, strength, speed, stamina, etc. The pre-season period lasted eight weeks. The microcycle training schedule for pre-season is presented in Table 1. In alignment with the specific focus of this study, the data used for analysis pertained to the training sessions.

**Table 1:** Microcycle training schedule during pre-season.

	Monday			Tuesday			Wednesday			Thursday			Friday			Saturday	Sunday
	(Acceleration-deceleration day)			(Stamina day)						(Speed day)			(High-intensity day)				
	Training	Intensity	Duration	Training	Intensity	Duration	Training	Intensity	Duration	Training	Intensity	Duration	Training	Intensity	Duration		
Morning	Strength lower body	-80 to 95%	60 min	Collective rugby with a high volume of running		110 min	Upper body hypertrophy		60 min	Long sprint	High intensity	60 min	Full tackle	Try to have the intensity as a match		Off	
		-3 blocks															
		-2∼5 repetitions															
		-4 sets															
	Install rugby	Low intensity work on the strategic part	45 min				Recovery on ice bath		45 min	Upper and lower body power session		60 min	Short training		50 min		
Afternoon	Rugby working on acceleration	Short space-loss of short sprint	70 min	Upper body strength	-80 to 95%	60 min	Working on breath session with a freediver			Install rugby and contacts training	Low intensity work on the strategic part	30 min + 15 min					
					-3 blocks												
					-2∼5 Repetitions												
					-4 sets												
				Wrestling-specific rugby session		40 min											

### 2.3 Workload monitoring

#### 2.3.1 GPS-derived workload measures

Workload monitoring was conducted using GPS devices (Vector X7 sensors, Catapult Innovations, Australia) which included 10 Hz GPS, 100 Hz triaxial accelerometer, gyroscope, and 100 Hz magnetometer. Each player was equipped with a specially designed vector garment, housing the sensor, positioned at the upper back between the shoulder blades. The validity and reliability of this equipment for monitoring running and mechanical measures in team sports have been substantiated in previous studies [33,34]. The GPS and inertial data were exported using a specialized GPS software (Openfield Console 3.7) to facilitate further analysis. Player workload data were reported as the total distance (TD) covered, player load [PL, arbitrary unit (AU)], counts of accelerations and decelerations above 2.5 m s^−2^ (ACC_num_ + DEC_num_ >2.5 m s^−2^), total number of accelerations above 2.5 m s^−2^ (ACC_num_ >2.5 m s^−2^), total accelerations distance above 2 m s^−2^ (AD >2 m s^−2^), high-speed running distance (HSR, speed >15 km h^−1^) [35,36], very high-speed running distance (VHSR, speed >21 km h^−1^) [35,36], and sprint running distance (SR, speed >25 km h^−1^) [37,38]. The PL was calculated using the instantaneous PL formula [39,40]:

$$Player\;Load=\sqrt{\left({\left(fwd_{t=i+1}-fwd_{t=i}\right)}^{2}+{\left({side}_{t=i+1}-{side}_{t=i}\right)}^{2}+{\left(up_{t=i+1}-up_{t=i}\right)}^{2}\right)}$$

Where fwd: forward acceleration; side: sideways acceleration; up: upwards acceleration; t: time.

Accumulated player load formula [41,42]:

$$Player\;Load\;{\left(acc\right)}_{t=n}=\sum_{t=0}^{t=n}\sqrt{\left({\left(fwd_{t=i+1}-fwd_{t=i}\right)}^{2}+{\left({side}_{t=i+1}-{side}_{t=i}\right)}^{2}+{\left(up_{t=i+1}-up_{t=i}\right)}^{2}\right)}$$

for t = 0, 0.01, 0.02, 0.03 … n.

#### 2.3.2 Three methods for calculating ACWR

**Fig. 1:**
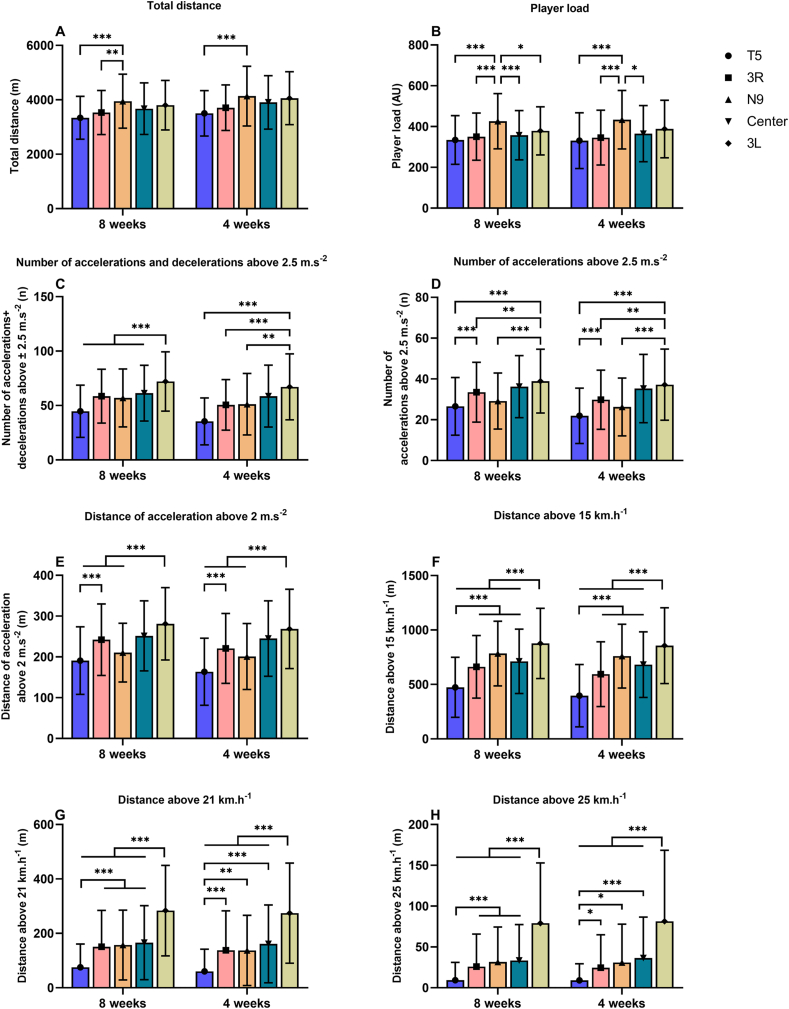
Comparison of eight-week and last four-week workload of players during pre-season in five positions. *p < 0.05, **p < 0.01, ***p < 0.001.

Given that the chronic workload spans over twenty-eight days, our analysis focused on calculating ACWR from week five through eight, which corresponds to the four weeks illustrated in Fig. 1. The ACWR for workload evaluation was determined by employing three distinct methods: coupled RA, uncoupled RA, and EWMA. The coupled RA ACWR is calculated as the workload of the current week divided by the average weekly workload of the previous four weeks. The corresponding formula was: coupled RA = total workload for the current week/0.25*(total workload for the current week + W1+W2+W3). W1, W2 and W3 represent the workload for the preceding three weeks, respectively [24]. On the other hand, the uncoupled RA method utilized in this research involves dividing the acute workload for the current week (represented by one-week workload) by the average workload of the previous three weeks, which is considered as the chronic workload. The formula was: uncoupled RA = total workload for the current week/0.333*(W1+W2+W3) [[43], [44], [45]].

For a given day, the EWMA was calculated as follows: ${EWMA}_{today}={workload}_{today}\times {ƛ}_{a}+\left[\left(1-{ƛ}_{a}\right)\times {EWMA}_{yesterday}\right]$ [46]. In this formula, ${ƛ}_{a}$ (workload decay rate) was calculated as 2/(N+1), with values ranging from 0 to 1. In this study, the chosen time decay constant, N, with N=7 used to represent acute workload and N=28 used to represent chronic workload [47]. Consequently, the formula for acute workload became ${EWMA}_{today}={workload}_{today}\times \frac{2}{7+1}+\left[\left(1-\frac{2}{7+1}\right)\times {EWMA}_{yesterday}\right]$, and the formula for chronic workload was ${EWMA}_{today}={workload}_{today}\times \frac{2}{28+1}+\left[\left(1-\frac{2}{28+1}\right)\times {EWMA}_{yesterday}\right]$. The EWMA ACWR for a given day was the ratio of the above two (acute:chronic).

### 2.4 Statistical analysis

All statistical analyses were conducted using the GraphPad Prism (GraphPad Software 9), with the level of significance set at p < 0.05. Mean ± SD was calculated as descriptive statistics. The Shapiro-Wilk test and the visualization of normality plots were used to make assumptions of the data's normality prior to analysis. Measures of pre-season period workload and comparison of three ACWR methods in the same measure and the same playing position were analyzed using one-way analysis of variance (ANOVA) with Tukey's multiple comparisons test. Two-way ANOVA was used when comparing differences among three ACWR methods in the same measure for players in different playing positions. Cohen's d was calculated to measure the effect size (ES) statistic between two groups. ES of ≤0.20, 0.21–0.6, 0.61–1.20, 1.21–2.0 and ≥ 2.0 were used to represent trivial, small, moderate, large, and very large effects, respectively [48].

## 3 Results

### 3.1 Effects of the playing position

Fig. 1 presents a comparison between the overall eight-week pre-season workload and the workload over the final four-weeks for players across the five distinct positions. The trend of workload over the last four weeks across different playing positions was consistent with observations throughout the entire eight-week pre-season period. Across the pre-season period, N9 exhibited higher workload than T5 and 3R in terms of TD (N9: 3274.35 ± 907.32 m, p < 0.01, ES = 0.68 and 0.46) and PL (N9: 368.82 ± 119.78 AU, p < 0.001, ES = 0.16 and 0.61) (Fig. 1A and B). Meanwhile, 3L players had the highest workload in ACC_num_ + DEC_num_ (60.74 ± 26.74, p < 0.01, ES = 1.06, 0.52, and 0.5), ACC_num_ (241.2 ± 96.65, p < 0.01, ES = 0.83, 0.36, and 0.66), AD (60.74 ± 26.74, p < 0.01, ES = 1.05, 0.44, and 0.87), HSR (741.23 ± 364.19 m, p < 0.01, ES = 1.35, 0.71 and 0.3), VSHR (237.39 ± 170.05 m, p < 0.01, ES = 1.58, 0.88, and 0.85) and SR (67.62 ± 71.21 m, p < 0.01, ES = 1.28, 0.89, and 0.79) in comparison to T5, 3R, and N9 (Fig. 1C–H).

### 3.2 Effects of the calculation method

**Fig. 2:**
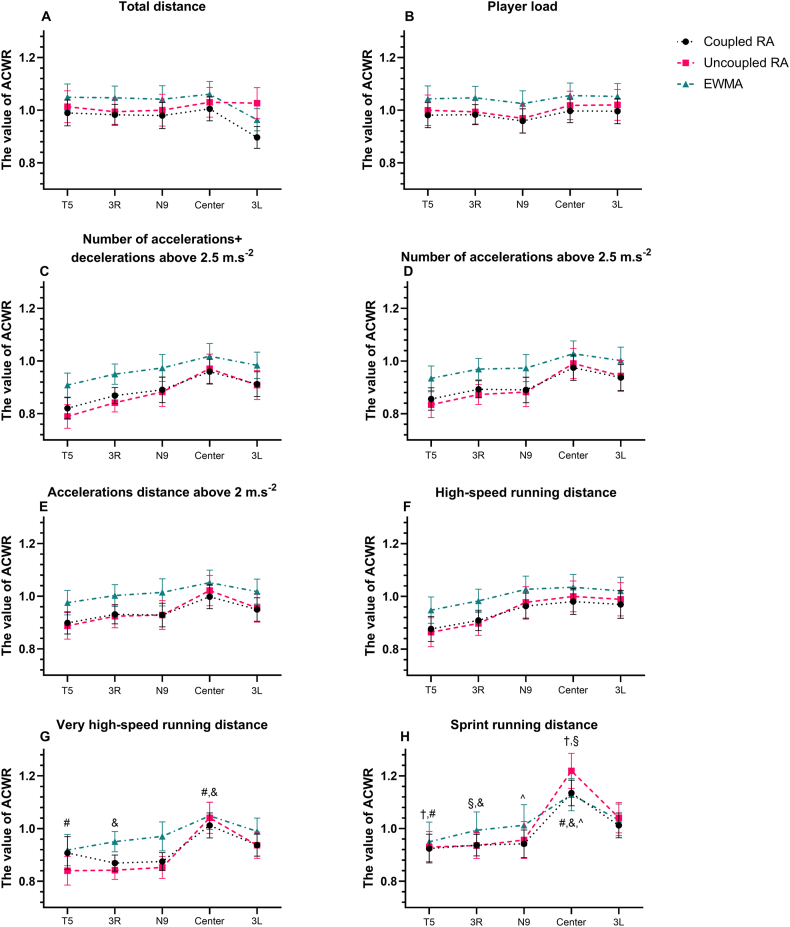
Comparison of the differences between the three ACWR methods in five positions. †: T5 *vs.* center, coupled RA method, p < 0.05; §: 3R *vs.* center, coupled RA method, p < 0.05; #:T5 *vs.* center, uncoupled RA method, p < 0.001; &: 3R *vs.* center, uncoupled RA method, p < 0.001; ^: N9 *vs*. center, uncoupled RA method, p < 0.01.

Table 2 provides a comprehensive comparison of the three ACWR methods across identical measures and playing position. Specifically, the ACWR derived from the uncoupled RA was found to be higher than that from the coupled RA for TD in 3L (p < 0.05, ES = 0.48). For ACC_num_ + DEC_num_ and ACC_num_, both the T5 and 3R groups had the lowest ACWR values for uncoupled RA, the highest calculated values obtained by EWMA, and there were significant differences (p < 0.05, ES = 0.11–0.56) between the three methods. This situation also occurred in 3R and N9 (p < 0.05, ES = 0.1–0.56) for VHSR. For SR, uncoupled RA was significantly higher (p < 0.05, ES = 0.26) than coupled RA in centers. In addition to comparing the differences between the three methods, differences were also observed among five groups for the same method. When using coupled RA method, the centers were exposed to greater values for SR than T5 (p < 0.05, ES = 0.8) and 3R (p < 0.05, ES = 0.83) (Fig. 2H). In addition, when the uncoupled RA method used, the centers exhibited greater ACWR values for VHSR (p < 0.05, ES = 0.67 and 0.69) (Fig. 2G) and SR (p < 0.05, ES = 0.86 and 0.91) compared to T5 and 3R (Fig. 2H). Regarding the other metrics, no significant differences were observed between the groups by the same ACWR method (Fig. 2A–F).

**Table 2:** Mean (±SD) value for comparison of three ACWR methods between the same measures and the same position.

	T5			Cohen's *d*-effect		
Workload measures	Coupled RA	Uncoupled RA	EWMA	Coupled RA *vs.* Uncoupled RA	Coupled RA *vs.* EWMA	Uncoupled RA *vs*. EWMA
TD (m)	0.99 ± 0.26	1.01 ± 0.32	1.05 ± 0.27	0.07 (T)	0.23 (S)	0.14 (T)
PL (AU)	0.98 ± 0.25	1.00 ± 0.31	1.04 ± 0.26	0.07 (T)	0.24 (S)	0.14 (T)
ACC_num_ + DEC_num_ > 2.5 m s^−2^ (n)	**0.82 ± 0.21**^a^^**,**§^	**0.79 ± 0.24**^a^^**,**#^	**0.91 ± 0.24**^§**,**#^	0.13 (T)	0.40 (S)	0.50 (S)
ACC_num_ >2.5 m s^−2^ (n)	**0.86 ± 0.23**^a^	**0.83 ± 0.26**^a^^**,**#^	**0.93 ± 0.25**^#^	0.12 (T)	0.29 (S)	0.39 (S)
AD > 2 m s^−2^ (n)	0.90 ± 0.22	0.89 ± 0.27	0.98 ± 0.25	0.04 (T)	0.34 (S)	0.35 (S)
HSR (m)	0.88 ± 0.25	0.86 ± 0.29	0.95 ± 0.27	0.07 (T)	0.27 (S)	0.32 (S)
VHSR (m)	0.91 ± 0.33	0.84 ± 0.29	0.92 ± 0.32	0.19 (T)	0.03 (T)	0.26 (S)
SR (m)	0.92 ± 0.29	0.93 ± 0.32	0.95 ± 0.40	0.03 (T)	0.09 (T)	0.06 (T)
	3R			Cohen's *d*-effect		
	Coupled RA	Uncoupled RA	EWMA	Coupled RA *vs.* Uncoupled RA	Coupled RA *vs.* EWMA	Uncoupled RA *vs*. EWMA
TD (m)	0.98 ± 0.21	0.99 ± 0.25	1.05 ± 0.24	0.05 (T)	0.31 (S)	0.24 (S)
PL (AU)	0.98 ± 0.20	0.99 ± 0.24	1.05 ± 0.23	0.05 (T)	0.32 (S)	0.26 (S)
ACC_num_ + DEC_num_ > 2.5 m s^−2^ (n)	**0.87 ± 0.16**^a^	**0.84 ± 0.19**^a^^**,**#^	**0.95 ± 0.20**^#^	0.17 (T)	0.44 (S)	0.56 (S)
ACC_num_ >2.5 m s^−2^ (n)	**0.89 ± 0.17**^a^	**0.87 ± 0.20**^a^^**,**#^	**0.97 ± 0.22**^#^	0.11 (T)	0.41 (S)	0.48 (S)
AD >2 m s^−2^ (n)	0.93 ± 0.19	0.92 ± 0.23	1.00 ± 0.22	0.05 (T)	0.34 (S)	0.36 (S)
HSR (m)	0.91 ± 0.20	0.90 ± 0.24	0.98 ± 0.24	0.05 (T)	0.32 (S)	0.33 (S)
VHSR (m)	**0.87 ± 0.16**^a^	**0.84 ± 0.19**^a^^**,**#^	**0.95 ± 0.20**^#^	0.17 (T)	0.44 (S)	0.56 (S)
SR (m)	0.94 ± 0.22	0.94 ± 0.26	0.99 ± 0.37	0.00 (T)	0.16 (T)	0.16 (T)
	N9			Cohen's *d*-effect		
	Coupled RA	Uncoupled RA	EWMA	Coupled RA *vs.* Uncoupled RA	Coupled RA *vs.* EWMA	Uncoupled RA *vs*. EWMA
TD (m)	0.98 ± 0.26	1.00 ± 0.32	1.04 ± 0.27	0.07 (T)	0.23 (S)	0.14 (T)
PL (AU)	0.96 ± 0.24	0.97 ± 0.29	1.03 ± 0.26	0.04 (T)	0.28 (S)	0.22 (S)
ACC_num_ + DEC_num_ > 2.5 m s^−2^ (n)	0.89 ± 0.26	0.88 ± 0.3	0.97 ± 0.27	0.04 (T)	0.30 (S)	0.32 (S)
ACC_num_ >2.5 m s^−2^ (n)	0.89 ± 0.26	0.88 ± 0.29	0.97 ± 0.27	0.04 (T)	0.30 (S)	0.32 (S)
AD >2 m s^−2^ (n)	0.93 ± 0.24	0.93 ± 0.29	1.01 ± 0.27	0.00 (T)	0.31 (S)	0.29 (S)
HSR (m)	0.96 ± 0.26	0.98 ± 0.32	1.03 ± 0.27	0.07 (T)	0.26 (S)	0.17 (T)
VHSR (m)	**0.87 ± 0.18**^a^	**0.85 ± 0.22**^a^^**,**#^	**0.97 ± 0.29**^#^	0.10 (T)	0.41 (S)	0.47 (S)
SR (m)	0.94 ± 0.28	0.96 ± 0.37	1.01 ± 0.41	0.06 (T)	0.20 (S)	0.13 (T)
	center			Cohen's *d*-effect		
	Coupled RA	Uncoupled RA	EWMA	Coupled RA *vs.* Uncoupled RA	Coupled RA *vs.* EWMA	Uncoupled RA *vs*. EWMA
TD (m)	1.01 ± 0.24	1.03 ± 0.30	1.06 ± 0.26	0.07 (T)	0.20 (S)	0.11 (T)
PL (AU)	1.00 ± 0.24	1.02 ± 0.29	1.06 ± 0.25	0.08 (T)	0.24 (S)	0.15 (T)
ACC_num_ + DEC_num_ > 2.5 m s^−2^ (n)	0.96 ± 0.25	0.97 ± 0.29	1.02 ± 0.26	0.04 (T)	0.24 (S)	0.18 (T)
ACC_num_ >2.5 m s^−2^ (n)	0.97 ± 0.25	0.99 ± 0.3	1.03 ± 0.26	0.07 (T)	0.24 (S)	0.14 (T)
AD >2 m s^−2^ (n)	1.00 ± 0.24	1.02 ± 0.30	1.05 ± 0.25	0.07 (T)	0.20 (S)	0.11 (T)
HSR (m)	0.98 ± 0.26	1.00 ± 0.31	1.03 ± 0.26	0.07 (T)	0.19 (T)	0.10 (T)
VHSR (m)	1.01 ± 0.25	1.04 ± 0.31	1.05 ± 0.28	0.11 (T)	0.15 (T)	0.03 (T)
SR (m)	**1.14 ± 0.26**^a^	**1.22 ± 0.35**^a^	1.13 ± 0.32	0.26 (S)	0.03 (T)	0.27 (S)
	3L			Cohen's *d*-effect		
	Coupled RA	Uncoupled RA	EWMA	Coupled RA *vs.* Uncoupled RA	Coupled RA *vs.* EWMA	Uncoupled RA *vs*. EWMA
TD (m)	**0.9 ± 0.22**^a^	**1.03 ± 0.31**^a^	0.96 ± 0.22	0.48 (S)	0.27 (S)	0.26 (S)
PL (AU)	1.00 ± 0.25	1.02 ± 0.31	1.05 ± 0.26	0.07 (T)	0.20 (S)	0.10 (T)
ACC_num_ + DEC_num_ > 2.5 m s^−2^ (n)	0.91 ± 0.25	0.91 ± 0.29	0.98 ± 0.26	0.00 (T)	0.27 (S)	0.25 (S)
ACC_num_ >2.5 m s^−2^ (n)	0.94 ± 0.26	0.94 ± 0.31	1.00 ± 0.27	0.00 (T)	0.23 (S)	0.21 (S)
AD >2 m s^−2^ (n)	0.95 ± 0.24	0.96 ± 0.29	1.02 ± 0.25	0.04 (T)	0.29 (S)	0.22 (S)
HSR (m)	0.97 ± 0.28	0.99 ± 0.33	1.02 ± 0.28	0.07 (T)	0.18 (T)	0.10 (T)
VHSR (m)	0.94 ± 0.22	0.94 ± 0.27	0.99 ± 0.27	0.00 (T)	0.20 (S)	0.19 (T)
SR (m)	1.01 ± 0.25	1.04 ± 0.31	1.03 ± 0.32	0.11 (T)	0.07 (T)	0.03 (T)

ES: effect size; T: trivial effect; S: small effect; TD: total distance; PL: player load, ACC_num_ + DEC_num_ >2.5 m s^−2^: counts of accelerations and decelerations above 2.5 m s^−2^; ACC_num_ >2.5 m s^−2^: total accelerations above 2.5 m s^−2^; AD >2 m s^−2^: total accelerations distance above 2 m s^−2^; HSR: high-speed running distance (speed >15 km h^−1^); VHSR: very high-speed running distance (speed >21 km h^−1^); SR: sprint running distance (speed >25 km h^−1^). RA: rolling average; EWMA: exponentially weighted moving average; m: meter; n: number; AU: arbitrary unit.

a Coupled RA *vs*. Uncoupled RA in the same position, p < 0.05; §: Coupled RA *vs*. EWMA in the same position, p < 0.05; #: Uncoupled RA *vs*. EWMA in the same position, p < 0.05.

## 4 Discussion

The main evidence of the present study showed that N9 players exhibited the highest TD and PL, whereas 3L players demonstrated superior levels of acceleration-deceleration and running workload in different high-speed zones. In contrast, T5 players consistently had the lowest workload among the observed groups during pre-season. Notably, centers exhibited the highest ACWR values. Furthermore, when comparing the different ACWR methods, distinctions emerged among specific positions and measures using three models (e.g. in positions T5 and 3R, there were significant differences among the three methods in ACC_num_ + DEC_num_ and ACC_num_ measures). However, it is crucial to highlight that these differences, while statistically significant, were characterized by a low magnitude due to consistently low ES outcomes across all measures. These previous results serve to substantiate our initial hypothesis that (i) noticeable differences can be observed in workload and ACWR based on the playing positions of professional RU players, and (ii) the quantification of pre-season workload by the three models does not differ considerably.

### 4.1 Playing position differences

The established influence of playing position on match demands in team sports underscores discernible disparities in physical requisites [11,49]. Players in the Australian Rugby League were classified into four positional groups: hit-up forwards (props and lock), wide-running forwards (second rowers), adjustables (hookers, halfbacks, five-eighths, and fullbacks), and outside backs (centers and wingers). Within these groups, hit-up forwards covered the least distance during matches, followed by wide-running forwards, adjustables, and outside backs, who covered the longest distance [50]. In RU, diverging from the customary delineation of positions into forwards and backs. Our findings align with previous research emphasizing discernible movement demands across different positional clusters. These studies indicated that over an eight-week pre-season period, measures pertaining to TD, acceleration, and high-speed running zones were elevated for backs when compared to forwards [[51], [52], [53]]. In the research of Cahill et al. [54], player positions in RU were defined as front, second and back rows, scrum-half, inside and outside backs. They discovered that the scrum-half covered the greatest TD, while the front and second rows (T5) covered the least during a match. Our analysis also unveils the same results. This can be attributed to the active role assumed by this position, involving pace management and organizing the team's offensive maneuvers with the aim of exerting greater influence over the match's trajectory, necessitating increased running and shifting movements than other positions [51,55]. The evidence indicating that the T5 covered the least distance aligns with the notion that their primary roles center around contesting possession at set-pieces and break-downs. In performing these duties, they engage in more static activities such as tackling, scrimmaging, and rucking compared to backs [56].

This study also found that N9 players had a significantly higher PL than other positions. This might be explained by the fact that they result from both being tackled and making sudden, fleeting direction changes during games [49]. However, in Roe et al.'s study, the disparity in PL between forwards and backs was minimal. This discrepancy may be attributed to variations in player age, playing tactics or strategies across different countries [57]. In accordance with previous research [[58], [59], [60], [61]], when considering measures such as accelerations, decelerations, and distance covered in different high-speed zones, backs (especially the outside backs) exhibited superior values compared to forwards. This discrepancy arises from backs covering greater VHSR, SR, and exhibiting more frequent accelerations and decelerations than their forward counterparts during matches, thereby demanding a higher frequency of high-intensity activities [62,63].

Furthermore, our results showed that centers exhibited a higher ACWR values across all measures, aligning with previous research in team sports like football. Oliveira et al. showed meaningfully higher coupled RA ACWR values for HSR in central midfielders [64]. These differences underscore the distinctive athletic profiles of professional RU players in specific positional roles, emphasizing the importance of individualized training programs.

### 4.2 ACWR calculation differences

The widespread utilization of ACWR serves as an invaluable tool for coaches, providing a detailed insight into each player's fatigue and fitness status [26]. While previous research has employed these methods to quantify workload in team sports [44,65], no studies to date have yet been reported to quantify the differences of three ACWR methods during the pre-season period for RU. Hulin et al. have concluded that there is no difference between RA and EWMA methods [66]. Our study aligns with this consensus. In some cases (e.g., when comparing ACC_num_ + DEC_num_ and ACC_num_ parameters for 3L position players), the three calculations appeared to be significantly different. However, when ESs were calculated, all results were found to be small (<0.6). These outcomes further corroborate Coyne's research, which demonstrated trivial differences and a strong correlation between coupled and uncoupled ACWR due to the minimal effects [67].

Given the distinct characteristics of the three methods used for calculating ACWR, coupled and uncoupled RA method have gained widespread acceptance for its simplicity and flexible calculation. In addition, the EWMA model places particular emphasis on calculating workload at the conclusion of distinct training periods, such as seven-day or twenty-eight-day intervals. The temporal aspect of the EWMA model becomes evident as the initial workload gradually diminishes in influence on the current workload day by day over time. This characteristic renders the EWMA model especially well-suited for portraying training schedules in the near future [31,47]. Therefore, despite the differences in calculation methods among the three models, the results remain notably similar. We advocate for practitioners in this field to choose any of these three approaches.

### 4.3 Study limitations

While this study introduces a novel perspective, it is important to acknowledge several inherent limitations. Firstly, given our primary focus on evaluating pre-season workload, the availability of ACWR data was limited to a mere twenty-eight days. This constraint should be addressed in future studies which explore the impact of pre-season training on the in-season period. Secondly, this study pertained to the absence of formal validity and reliability testing for the Vector X7 device, which may raise concerns about the accuracy and consistency of the gathered data. Future research should prioritize a thorough assessment of this to improve the suitability of the device for research applications. Finally, it is essential to recognize that ACWR is not without constraints, particularly in coupled ACWR, the repeated calculation of the impact of acute workload may result in a biased estimation of the actual effects [68,69]. Additionally, normalizing the ratio of acute to chronic workload within a restricted range has the potential to confound the true level of acute workload [70]. By restricting the range, we may overlook extreme workload variations that could be relevant to an athlete's performance or injury risk. Future research should investigate the validity and reliability of quantifying workload using extensive datasets.

## 5 Practical applications

Our study findings offer practical implications for coaches operating during the pre-season phase. Utilizing the results as a reference point, coaches and training staff can effectively monitor positional workload variations during this critical period. Considering that the workload for forwards is low (especially T5), coaches should focus on improving overall fitness. This can be achieved by exposing the forward groups to a series of high-intensity running protocols that allow them to engage in extensive or prolonged running at a higher workload. Also, implement acceleration and deceleration training strategies to help players manage accelerated power. For the backs, running efficiency needs to be maximized by focusing on the ability to accelerate and decelerate safely and efficiently from high speeds. The emphasis should be on covering a velocity range with high quality. These enable a thorough assessment of player readiness, thereby facilitating the tailoring of training regimens to align with the unique attributes of players in different positions, in accordance with upcoming match demands. Additionally, when applying the ACWR method for workload quantification, coaches have the flexibility to choose from one of the three calculation methods available.

## 6 Conclusions

The current findings provide evidence in two key respects. Firstly, they affirm the equivalence of the ACWR calculation methods, namely coupled and uncoupled RA, as well as the EWMA. Secondly, they shed light on the demands of professional RU pre-season training sessions, demonstrating that N9 players were required to cover more total running distance and PL, while 3L players are called upon to execute higher levels of acceleration, deceleration and high-speed running workload.

## Ethics statement

All players were provided informed consent. The study was conducted in compliance with the Helsinki Declaration and followed the ethical guidelines of Heliyon. Furthermore, the study adhered to local legislation and institutional requirements and approved by the Ethics Committee of Rennes 2 University (approval number 2024-025).

## Data availability statement

The data that support the findings of this study are available on request from the corresponding author.

## CRediT authorship contribution statement

**Xiangyu Ren:** Writing – review & editing, Writing – original draft, Methodology, Investigation. **Simon Boisbluche:** Methodology, Data curation. **Kilian Philippe:** Writing – review & editing. **Mathieu Demy:** Software, Data curation, Conceptualization. **Xiaopan Hu:** Methodology. **Shuzhe Ding:** Supervision. **Jacques Prioux:** Writing – review & editing, Supervision.

## Declaration of competing interest

The authors declare that they have no known competing financial interests or personal relationships that could have appeared to influence the work reported in this paper.
